# Supplementary material for: Burnout Syndrome and Related Factors in Mexican Police Workforces
Source: Int J Environ Res Public Health. 2022 May 3;19(9):5537. doi: 10.3390/ijerph19095537 (PMC9102065; doi:10.3390/ijerph19095537)

Artificial Neuronal Network Outcomes.

Network Information			
**Input Layer**	Factors	1	Chronic diseases
		2	Hypertension
		3	Diabetes
		4	Digestive disorders
		5	Health Status
		6	Quality of diet
		7	Regular mealtimes
		8	Sleep hours
	Covariates	1	BMI
		2	Age
		3	Seniority
	Number of Unitsa		26
	Rescaling Method for Covariates		Standardized
**Hidden Layer(s)**	Number of Hidden Layers		2
	Number of Units in Hidden Layer 1a		5
	Number of Units in Hidden Layer 2a		4
	Activation Function		Hyperbolic tangent
**Output Layer**	Dependent Variables	1	BO
	Number of Units		2
	Activation Function		Identity
	Error Function		Sum of Squares
a Excluding the bias unit			

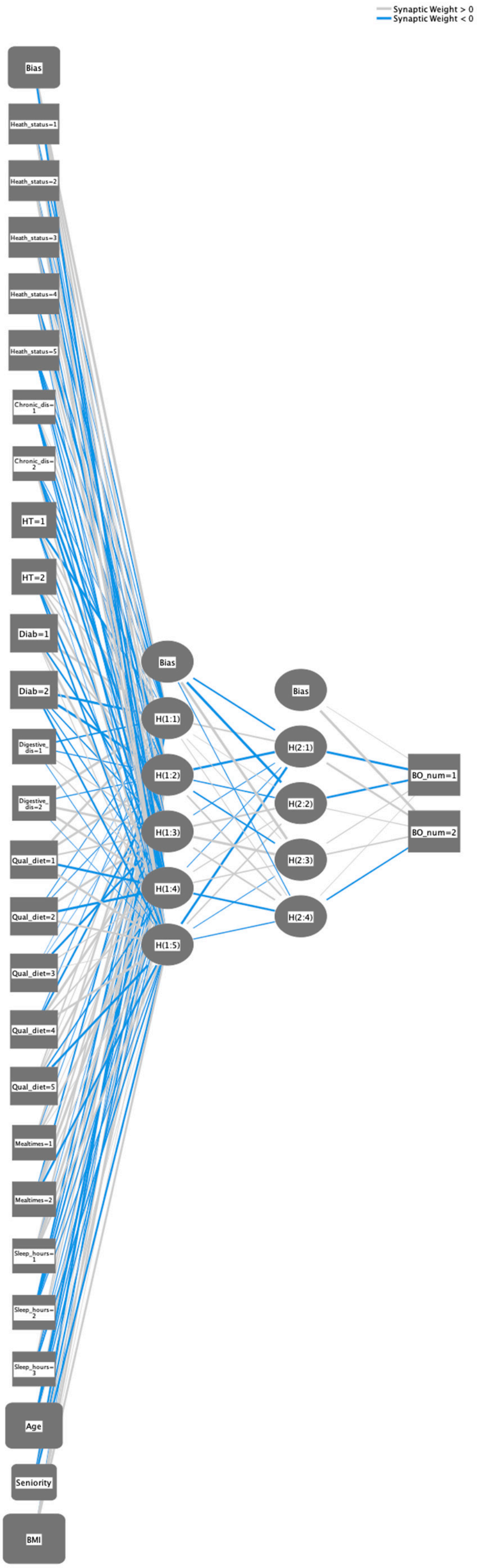

Hidden layer activation function: Hyperbolic tangent

Output layer activation function: Identity

Model Summary		
**Training**	Sum of Squares Error	24.626
	Percent Incorrect Predictions	18.70%
	Stopping Rule Used	1 consecutive step(s) with no decrease in error a
	Training Time	00:00.1
**Testing**	Sum of Squares Error	7.403
	Percent Incorrect Predictions	15.20%
**Dependent Variable: BO**		
a Error computations are based on the testing sample.		

Classification				
		Predicted		
**Sample**	**Observed**	***Risk***	***Normal***	***Percent Correct***
**Training**	Risk	13	26	33.30%
	Normal	6	126	95.50%
	Overall Percent	11.10%	88.90%	81.30%
**Testing**	Risk	3	10	23.10%
	Normal	0	53	100.00%
	Overall Percent	4.50%	95.50%	84.80%
**Dependent Variable: BO**				

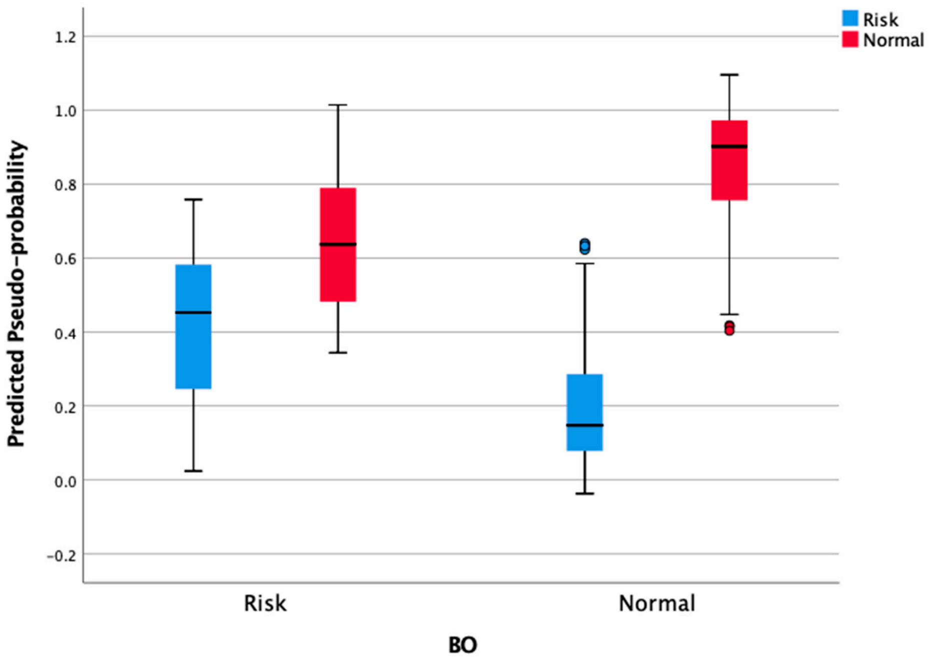

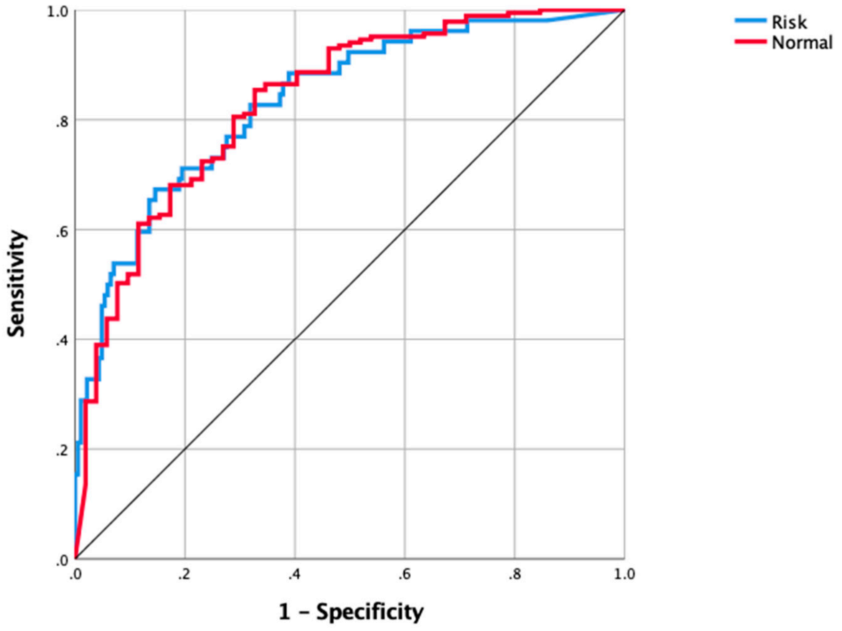

Dependent Variable: BO

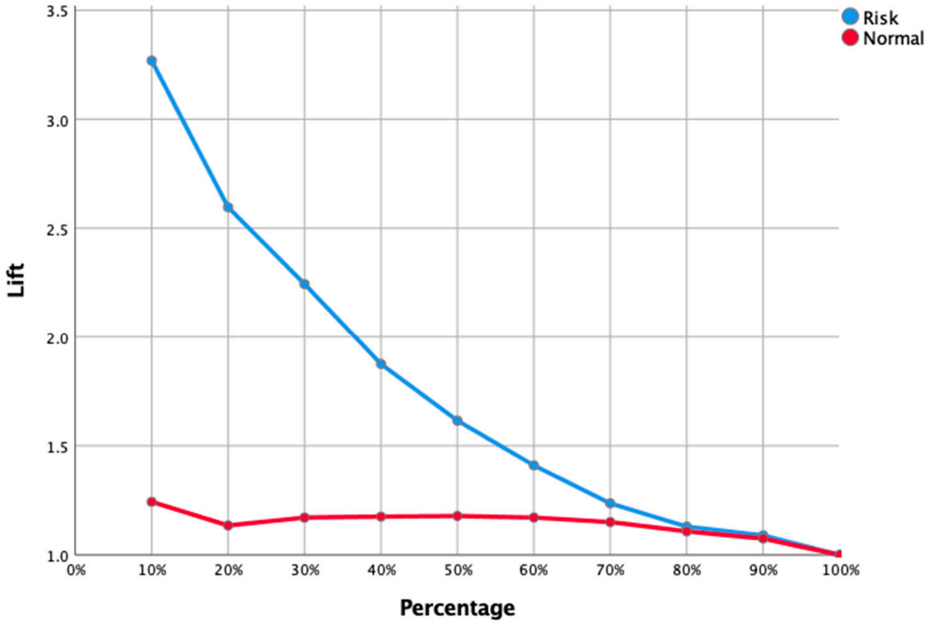

Dependent Variable: BO

Independent Variable Importance		
	Importance	Normalized Importance
**Health Status**	0.127	43.00%
**Chronic diseases**	0.015	5.20%
**Hypertension**	0.043	14.40%
**Diabetes**	0.084	28.30%
**Digestive disorders**	0.03	10.10%
**Quality of diet**	0.084	28.30%
**Regular mealtimes**	0.03	10.00%
**Sleep hours**	0.02	6.80%
**Age**	0.223	75.40%
**Seniority**	0.051	17.10%
**BMI**	0.295	100.00%

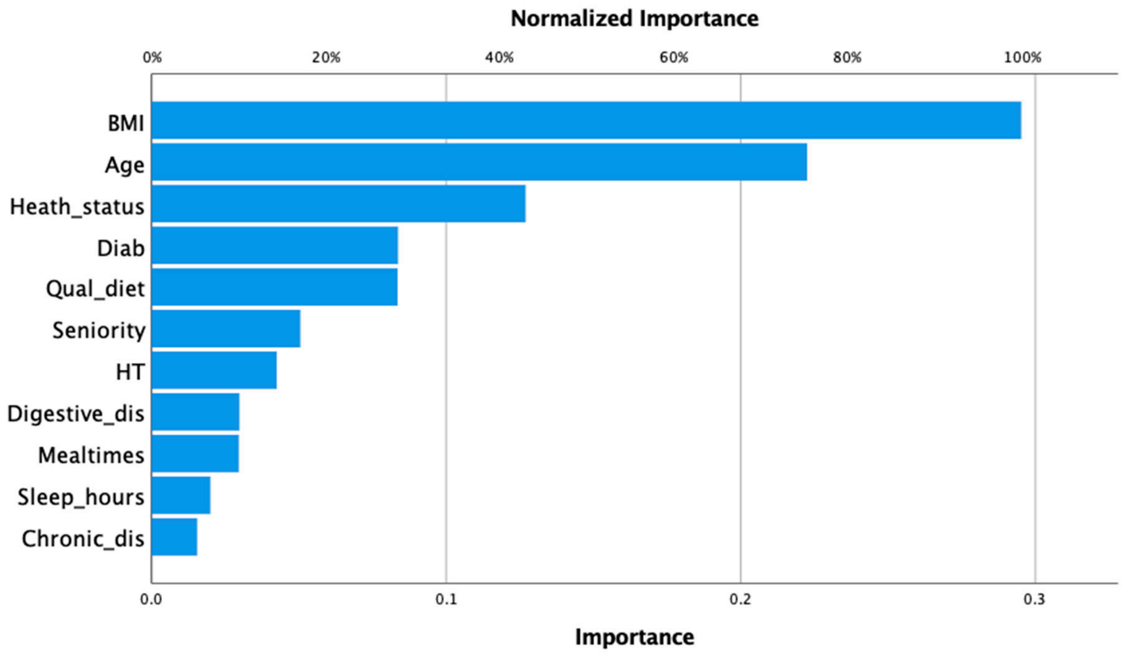

Supplement: Supplementary file 1 [file ijerph-19-05537-s001.zip › Artificial Neuronal Network Outcomes.pdf]
